# Development and validation of a nomogram for predicting immune‐related pneumonitis after sintilimab treatment

**DOI:** 10.1002/cam4.6708

**Published:** 2024-01-12

**Authors:** Baohui Hong, Rong Chen, Caiyun Zheng, Maobai Liu, Jing Yang

**Affiliations:** ^1^ Department of Pharmacy The Second Hospital of Sanming City Sanming China; ^2^ Department of Pharmacy Fujian Medical University Union Hospital Fuzhou China; ^3^ The School of Pharmacy Fujian Medical University Fuzhou China; ^4^ Department of Anesthesiology The Second Hospital of Sanming City Sanming China; ^5^ Fuqing City Hospital Affiliated to Fujian Medical University Fuzhou China

**Keywords:** immune checkpoint inhibitor, immune‐related adverse events, pneumonitis, risk factors, sintilimab

## Abstract

**Background:**

Immune‐related pneumonitis is a rare and potentially fatal adverse event associated with sintilimab. We aimed to develop and validate a nomogram for predicting the risk of immune‐related pneumonitis in patients treated with sintilimab.

**Methods:**

The least absolute shrinkage and selection operator (LASSO) regression was used to determine risk factors. Multivariable logistic regression was used to establish a prediction model. Its clinical validity was evaluated using calibration, discrimination, decision, and clinical impact curves. Internal validation was performed against the validation set and complete dataset.

**Results:**

The study included 632 patients; 59 were diagnosed with immune‐related pneumonitis. LASSO regression analysis identified that the risk factors for immune‐related pneumonitis were pulmonary metastases (odds ratio [OR], 4.015; 95% confidence interval [CI]: 1.725–9.340) and metastases at >3 sites (OR, 2.687; 95% CI: 1.151–6.269). The use of combined antibiotics (OR, 0.247; 95% CI: 0.083–0.738) and proton pump inhibitors (OR, 0.420; 95% CI: 0.211–0.837) were protective factors. The decision and clinical impact curves showed that the nomogram had clinical value for patients treated with sintilimab.

**Conclusions:**

We have developed and validated a practical nomogram model of sintilimab‐associated immune‐related pneumonitis, which provides clinical value for determining the risk of immune‐related pneumonitis and facilitating the safe administration of sintilimab therapy.

## Graphical abstract

Immune‐related pneumonitis is an adverse reaction to sintilimab immunotherapy, putting patients at risk for respiratory failure and death. This is the first risk prediction model incorporating relevant drug use factors for sintilimab‐induced immune‐related pneumonitis in patients with tumors. Our nomogram model may help clinicians assess the risk of immune‐related pneumonitis and develop optimal drug delivery strategies.

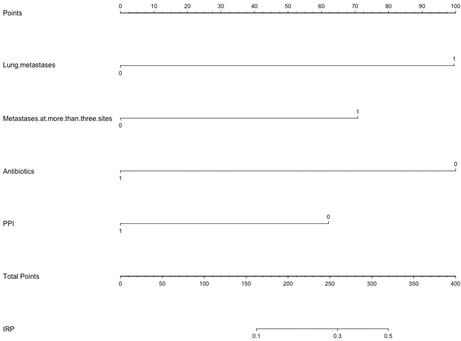

## 1 INTRODUCTION

Since the advent of immunotherapy, excellent antitumor efficacy has been observed with programmed death receptor‐1 (PD‐1) treatment in an increasing number of patients with tumors. Sintilimab is a recombinant human immunoglobulin G (IgG4)‐type programmed death receptor‐1 inhibitor developed for treating various solid tumors.^1^ A tumor immune response is generated by sintilimab via binding to PD‐1 and blocking the binding of PD‐1 to programmed death‐ligand 1 (PD‐L1) and PD‐L2 thus removing the immunosuppressive effect, activating the function of T cells, and enhancing the immune surveillance and tumor‐killing ability of T cells.^2^ However, the overactivation of T cells release an array of inflammatory cytokines, leading to immune‐related adverse reactions.^3^ Immune‐related pneumonitis (IRP) is a rare adverse reaction that usually causes nonspecific respiratory symptoms and parenchymal lung changes, which can progress to respiratory failure and even death.^4^ Clinical trials have reported that the incidence of IRP caused by sintilimab is between 3.4% and 4.3%.^5^, ^6^, ^7^ However, real‐world data suggest that the incidence of IRP is underestimated (10%–20%).^8^, ^9^ Therefore, an exploration of predictive factors for IRP occurrence is particularly important.

Early screening of high‐risk patients through predictive factors helps to monitor the occurrence of IRP, enabling prompt initiation of glucocorticoid therapy, and reducing the severity and mortality associated with IRP. In addition, predictors can affect prolonged survival and help identify patients who might benefit from cautious sintilimab therapy and those who do not complete the treatment. However, given the tendency of IRP to recur, restarting sintilimab treatment could further harm patients and increase financial costs.

The risk and severity of IRP is influenced by several factors, including low serum albumin levels, prior chest radiotherapy, and prior lung disease.^10^, ^11^, ^12^ However, the cessation of immune checkpoint inhibitor (ICI) treatment based solely on a single risk factor may be unreasonable and could adversely affect patients. Therefore, several clinical risk‐prediction models have been developed. For example, Jia et al. and Chao et al. have published nomograms predicting checkpoint inhibitor pneumonitis in non‐small cell lung cancer in 2022.^13^, ^14^ However, these studies were limited to non‐small cell lung cancer patients and included patients receiving different ICIs simultaneously. Therefore, no sintilimab‐induced IRP risk‐prediction model has yet been designed that independently uses data from patients with tumors treated with sintilimab.

This study aimed to analyze risk factors for IRP in patients treated with sintilimab and build a risk‐scoring model that can help clinicians predict the risk for IRP at an early stage, initiate timely and appropriate treatment, prevent the occurrence of higher levels of pulmonary toxicity, and identify the factors that should be considered before initiating ICI treatment.

## 2 MATERIALS AND METHODS

### 2.1 Data collection

Data of patients treated with sintilimab at the Union Hospital Affiliated to Fujian Medical University from January 2015 to May 2022 were retrospectively collected, including patient characteristics (such as age, sex, and alcohol and smoking history), disease characteristics (tumor type, tumor surgical history, pulmonary metastasis, brain metastasis, and number of metastatic sites), characteristics of comorbidities (such as hypertension, diabetes, coronary heart disease, stroke, chronic obstructive pulmonary disease, hepatitis, and syphilis), previous medication history (e.g., chemotherapeutics, targeted drugs, and other PD‐1s), combination therapy (e.g., chemotherapy, targeted drugs, antimicrobials, sputum agents, anti‐asthmatics, glucocorticoids, proton pump inhibitors [PPIs], or pirfenidone), treatment regimen of sintilimab (cycle and single dose), and hematological examination results (routine blood testing, electrolytes, lactate dehydrogenase, liver function, kidney function, and thyroid function indicators). Laboratory index ratios were calculated using the following methods: platelet‐lymphocyte ratio (PLR) = platelet count (×10^9^ cells/L)/lymphocyte count (×10^9^ cells/L) and neutrophil‐to‐lymphocyte ratio (NLR) = absolute neutrophil count (×10^9^ cells/L)/lymphocyte count (×10^9^ cells/L). Clinical data on IRP were collected retrospectively and classified according to the Common Terminology Criteria for Adverse Events (CTCAE), version 4.0.^15^

The inclusion criterion for this study was having tumors and receiving sintilimab. The exclusion criteria were as follows: (a) age <18 years, (b) treatment with a combination of sintilimab and other ICIs, (c) dropping out during follow‐up, and (d) missing data. The study was conducted in accordance with the Declaration of Helsinki and was approved by the Ethics Committee of Fujian Union Medical College Hospital. Due to the retrospective nature of this study, the requirement for written informed consent was waived.

### 2.2 Definition of immune‐related pneumonitis

IRP was defined as focal or diffuse inflammation affecting the lungs' soft tissues after initiation of sintilimab treatment.^15^ The diagnostic criteria for IRP^16^, ^17^ were (a) lung imaging abnormalities (such as ground glass shadow, patch consolidation, and fiber strip shadow) shown by chest computed tomography (CT) after treatment with sintilimab; (b) exclusion of heart failure and pulmonary embolism by comparing enhanced CT, laboratory indicators, echocardiography, and electrocardiogram; (c) exclusion of pulmonary infections by laboratory indicators and efficacy of antibiotic use; and (d) exclusion of tumor progression by laboratory indicators and pathological results (per the Response Evaluation Criteria in Solid Tumors). In addition, the toxicity of the IRP cases was graded according to the National Cancer Institute CTCAE, version 4.0. Patients who did not have IRP or were alive during data analysis were reviewed at the last follow‐up.

### 2.3 Statistical analysis

Statistical analysis was performed using R version 4.2.2 and STATA version 17.0. For comparison, we divided the training and verification sets by random sampling at a ratio of 7:3. Continuous variables are presented as mean or median (range of quartiles), and we conducted an independent sample *t*‐test or Mann–Whitney *U* test as appropriate. Categorical variables are presented as frequencies (percentages) and were analyzed using Fisher's exact test. Variable selection was performed via the least absolute shrinkage and selection operator (LASSO) regression analysis through 10‐fold cross‐validation.^18^ The variance inflation factor (VIF) was used to determine whether multicollinearity existed between the variables. Variables with a VIF >10 were excluded. A line graph prediction model was developed using logistic regression, and its performance was estimated.^19^ Internal validation was performed using validation sets and the complete dataset. An area under the curve (AUC) >0.7 was used to judge the ability of line graph discrimination.^20^ The Hosmer–Lemeshow test was used to evaluate the fit of the model. Calibration curves were constructed to evaluate the degree of the model calibration. Decision curve analysis (DCA) was performed to evaluate the clinical practicability of the model. We evaluated the model performance of the subgroups according to age, sex, and cancer type. The model was developed and validated according to the Transparent Reporting of a Multivariable Prediction Model for Individual Prognosis or Diagnosis (TRIPOD) (Table S1)^21^ The glmnet package was used for the LASSO regression. Column lines were drawn using the RMS software package. The AUC was plotted using the Hmisc and ROCR packages. All statistical tests were two‐sided, and statistical significance was set at *p* < 0.05.

## 3 RESULTS

### 3.1 Patient characteristics

Overall, 632 patients met the study criteria, namely, 59 in the IRP group and 573 in the control group, resulting in an incidence rate of 9.34%. The median age was 58 years (range 51.0–65.0 years), and 65.7% were male. Of these patients, 77.4% received a combination of chemotherapy and sintilimab (Table 1).

**TABLE 1:** Characteristics of patients in all datasets.

	All dataset	Non‐IRP	IRP	*p*‐Value
	(*n* = 632)	(*n* = 573)	(*n* = 59)	
Demographics				
Sex (male)	415 (65.7%)	370 (64.6%)	45 (76.3%)	0.08
Age (years)	58.0 (51.0, 65.0)	58.0 (50.0, 65.0)	59.0 (54.0, 66.0)	0.28
Smoker	339 (53.6%)	307 (53.6%)	32 (54.2%)	1
Drinker	278 (44.0%)	246 (42.9%)	32 (54.2%)	0.1
*Tumor characteristic*				
Tumor type				
Lung cancer	203 (32.1%)	175 (30.5%)	28 (47.5%)	0.09
Esophageal carcinoma	130 (20.6%)	122 (21.3%)	8 (13.6%)	
Gastric cancer	118 (18.7%)	111 (19.4%)	7 (11.9%)	
Lymphatic carcinoma	77 (12.2%)	73 (12.7%)	4 (6.8%)	
Bowel cancer	17 (2.7%)	16 (2.8%)	1 (1.7%)	
Liver cancer	45 (7.1%)	39 (6.8%)	6 (10.2%)	
Others	42 (6.6%)	37 (6.5%)	5 (8.5%)	
Surgery history	124 (19.6%)	114 (19.9%)	10 (16.9%)	0.73
Metastasis of lung	106 (16.8%)	70 (12.2%)	36 (61.0%)	<0.01
Metastasis of brain	25 (4.0%)	22 (3.8%)	3 (5.1%)	0.72
Number of metastatic sites ≥3	145 (22.9%)	111 (19.4%)	34 (57.6%)	<0.01
Concomitant disease				
COPD	21 (3.3%)	19 (3.3%)	2 (3.4%)	1
Hypertension	16 (2.5%)	16 (2.8%)	0 (0.0%)	0.39
CHD	18 (2.8%)	16 (2.8%)	2 (3.4%)	0.68
DM	9 (1.4%)	8 (1.4%)	1 (1.7%)	0.59
Stroke	8 (1.3%)	8 (1.4%)	0 (0.0%)	1
Syphilis	12 (1.9%)	11 (1.9%)	1 (1.7%)	1
Hepatitis	12 (1.9%)	10 (1.7%)	2 (3.4%)	0.31
Prior medication				
Prior chemotherapy	340 (53.8%)	308 (53.8%)	32 (54.2%)	1
Prior other PD‐1	28 (4.4%)	23 (4.0%)	5 (8.5%)	0.17
Prior TTD	131 (20.7%)	117 (20.4%)	14 (23.7%)	0.61
Sintilimab therapy regimen				
Period	3.0 (2.0, 5.0)	3.0 (2.0, 5.0)	3.0 (2.0, 6.0)	0.16
Dose	200.0 (200.0, 200.0)	200.0 (200.0, 200.0)	200.0 (200.0, 200.0)	0.3
Combination therapy				
TTD	147 (23.3%)	130 (22.7%)	17 (28.8%)	0.33
Chemotherapy	489 (77.4%)	455 (79.4%)	34 (57.6%)	<0.01
Expectorant	162 (25.6%)	160 (27.9%)	2 (3.4%)	<0.01
Pirfenidone	15 (2.4%)	13 (2.3%)	2 (3.4%)	0.64
Antibiotics	232 (36.7%)	227 (39.6%)	5 (8.5%)	<0.01
Anti‐asthmatic	173 (27.4%)	169 (29.5%)	4 (6.8%)	<0.01
Glucocorticoids	199 (31.5%)	186 (32.5%)	13 (22.0%)	0.11
H2	48 (7.6%)	46 (8.0%)	2 (3.4%)	0.3
PPI	446 (70.6%)	422 (73.6%)	24 (40.7%)	<0.01
Baseline laboratory examination				
ANC	4.6 (3.2, 5.6)	4.6 (3.2, 5.5)	4.8 (3.3, 5.6)	0.59
LYM	1.5 (1.1, 1.8)	1.5 (1.1, 1.8)	1.5 (1.1, 1.8)	0.74
PLT	245.2 (188.5, 288.0)	245.2 (188.0, 289.0)	245.2 (189.0, 277.0)	1
TBil	13.0 (13.0, 13.0)	13.0 (13.0, 13.0)	13.0 (13.0, 13.0)	0.45
ALP	88.5 (72.0, 104.9)	88.0 (72.0, 104.9)	93.0 (72.0, 106.0)	0.43
ALT	30.8 (17.0, 30.8)	30.8 (17.0, 30.8)	30.8 (24.0, 30.8)	0.44
AST	39.9 (21.5, 39.9)	39.9 (21.0, 39.9)	39.9 (28.0, 39.9)	0.04
FT3	4.7 (4.7, 4.7)	4.7 (4.7, 4.7)	4.7 (4.7, 4.7)	0.6
FT4	12.6 (12.4, 12.6)	12.6 (12.4, 12.6)	12.6 (12.3, 12.6)	0.96
TSH	2.4 (1.7, 2.4)	2.4 (1.7, 2.4)	2.4 (1.6, 2.4)	0.74
NLR	3.2 (2.1, 4.4)	3.2 (2.1, 4.5)	3.2 (2.3, 3.9)	0.98
PLR	166.9 (123.4, 226.9)	166.9 (125.0, 226.9)	166.9 (115.5, 244.7)	0.46
Serum albumin	38.1 (35.4, 41.3)	38.1 (35.4, 41.0)	38.5 (35.7, 42.7)	0.1
Cr	67.3 (57.0, 75.0)	67.3 (57.0, 75.0)	67.3 (59.0, 78.0)	0.56
LDH	200.5 (162.0, 221.0)	198.0 (159.0, 220.5)	220.5 (174.0, 247.0)	<0.01
Liver dysfunction	211 (33.4%)	190 (33.2%)	21 (35.6%)	0.77
Renal dysfunction	39 (6.2%)	36 (6.3%)	3 (5.1%)	1
Thyroid dysfunction	50 (7.9%)	49 (8.6%)	1 (1.7%)	0.07
Electrolyte abnormality	179 (28.3%)	162 (28.3%)	17 (28.8%)	1
PRO abnormality	53 (8.4%)	46 (8.0%)	7 (11.9%)	0.32
Glucose abnormality	138 (21.8%)	124 (21.6%)	14 (23.7%)	0.74

Abbreviations: ALP, alkaline phosphatase; ALT, alanine aminotransferase; ANC, absolute neutrophil count; AST, aspartate transaminase; CHD, coronary heart disease; COPD, chronic obstructive pulmonary disease; Cr, creatinine; DM, diabetes mellitus; FT3, free triiodothyronine; FT4, free tetraiodothyronine; H2‐RA, H2‐receptor antagonist; LDH, lactate dehydrogenase; LYM, lymphocyte; NLR, neutrophils‐to‐lymphocyte ratio; PD‐1, programmed cell death protein 1; PLR, platelet‐lymphocyte ratio; PLT, platelets; PPI, proton pump inhibitor; PRO, urine protein; TBil, total bilirubin; TSH, thyroid‐stimulating hormone; TTD, targeted therapy drug.

**FIGURE 1:**
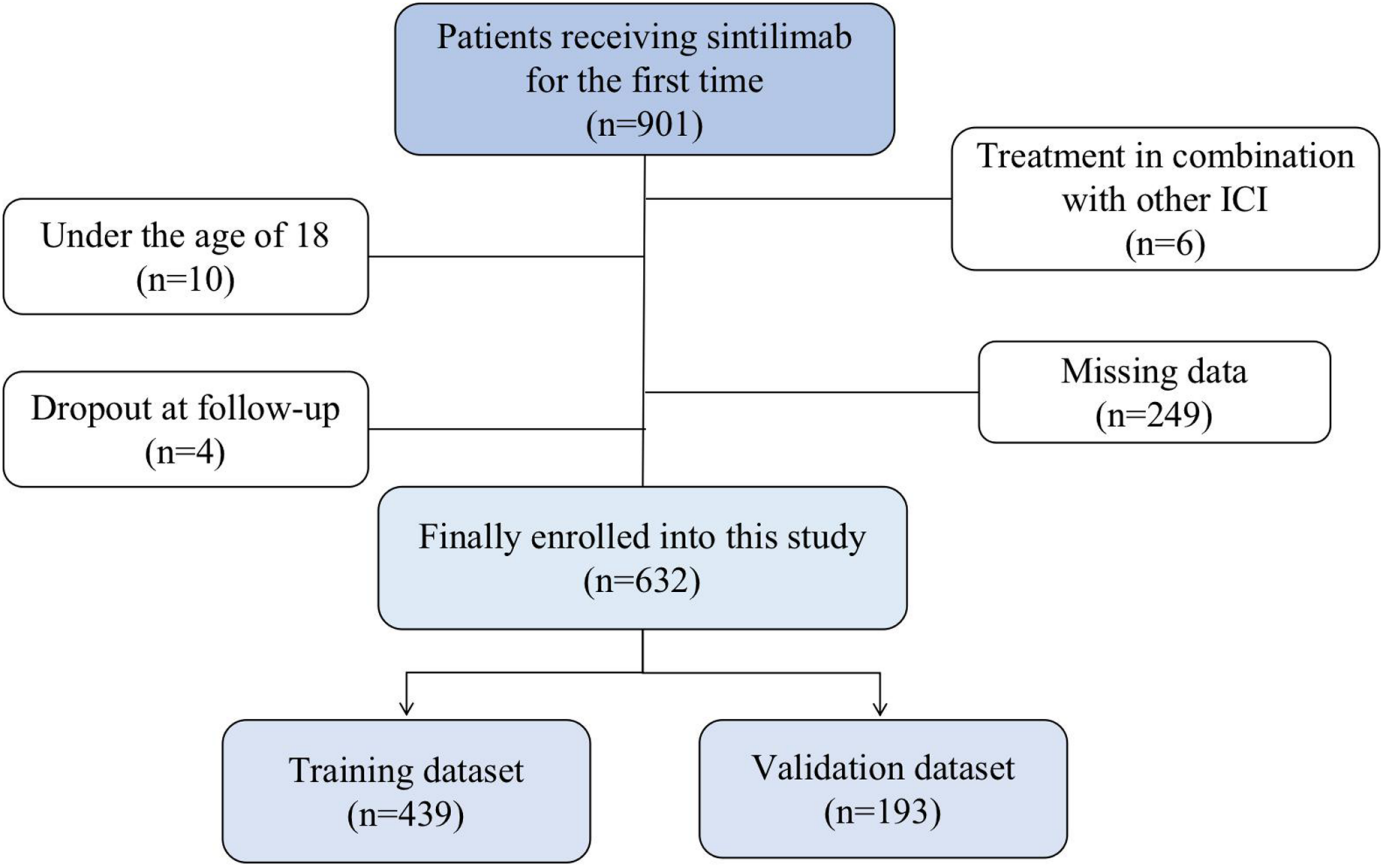
Flow diagram of data collection in this study. ICI, immune checkpoint inhibitor.

Through random sampling, 439 participants were included in the training set and 193 in the verification set (Figure 1). Table 1 shows that the incidences of lung metastases and metastases at ≥3 sites in patients with IRP were significantly different from those without IRP (*p* < 0.01). Baseline lactate dehydrogenase levels were considerably higher in patients with IRP than those without IRP (220.50 vs. 198.01 U/L, *p* < 0.01). Significant differences were observed in the distribution of combination chemotherapeutics, expectorants, antimicrobials, PPIs, or anti‐asthmatics between patients with IRP and those without IRP (*p* < 0.01).

### 3.2 Predictor selection

**FIGURE 2:**
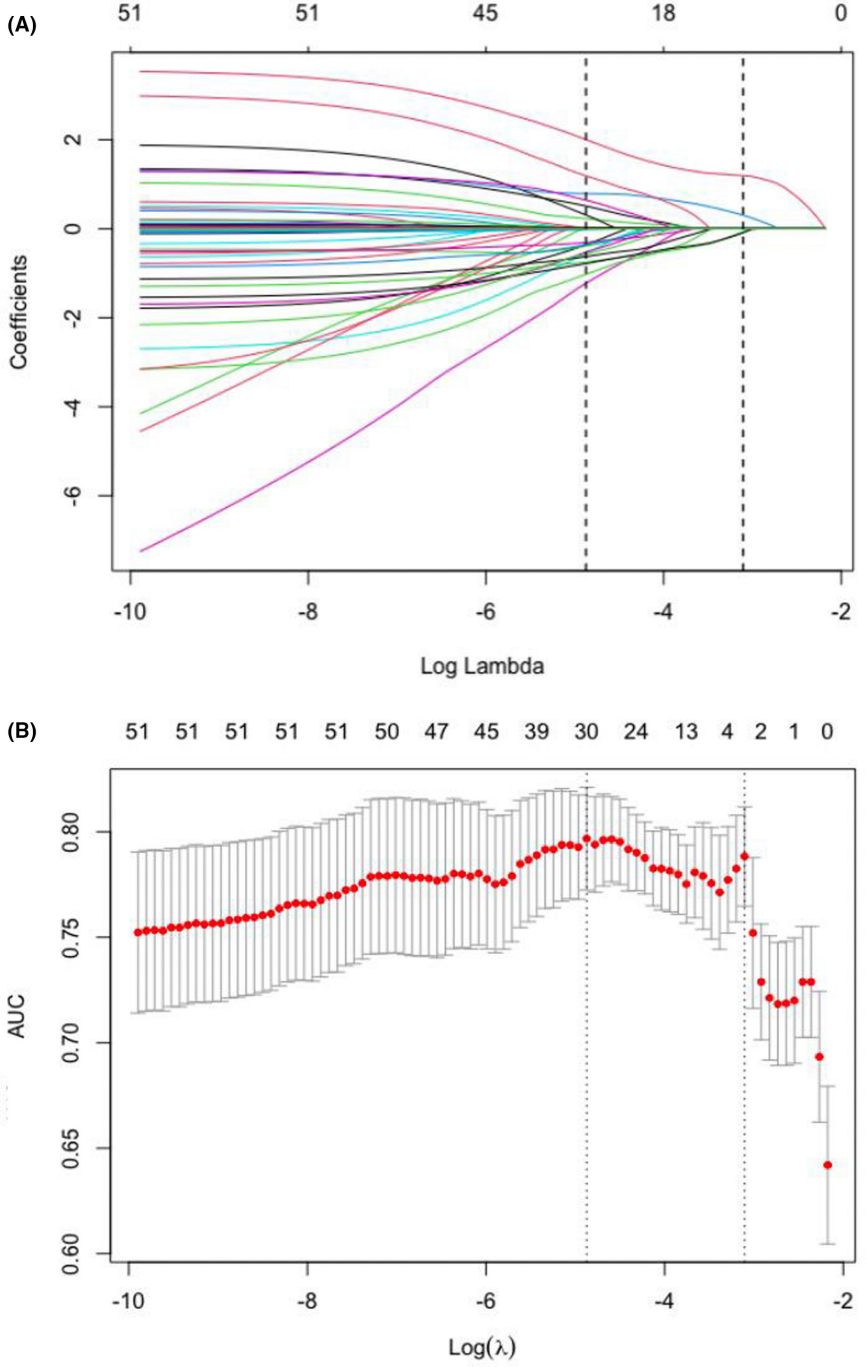
LASSO with a binary regression mode. (A) Least absolute shrinkage and selection operator coefficient distributions of 51 candidate predictors. (B) The area under the curve is denoted by the red points and error bars. The x‐axis is set at 10 times the adjustment penalty parameter log (λ) selected for cross‐verification. The left and right dotted vertical lines indicate the minimum and 1‐standard error criteria, respectively. The results show that four non‐zero coefficient predictors are selected.

Fifty‐one variables of patient characteristics were included in the LASSO regression analysis. After the LASSO regression selection (Figure 2 and Table S2), the four remaining clinical variables (lung metastases, metastases at ≥3 sites, combined antibiotic use, and combined PPI use) were factors for the final model to predict IRP risk. We tested multicollinearity in the prediction model to ensure the accuracy of the β‐coefficient and avoid false correlations and possible unreliable effect estimates. No multicollinearity was found according to the results of the collinearity test (Table S3). Therefore, the four variables were included in the logistic regression model, and the following results were obtained: lung metastasis (odds ratio [OR], 4.015; 95% confidence interval [CI]: 1.725–9.340) and metastases at ≥3 sites (OR, 2.687; 95% CI: 1.151–6.269) were associated with an increased risk of developing IRP, while combined antibiotic use (OR, 0.247; 95% CI: 0.083–0.738) and combined PPI use (OR, 0.420; 95% CI: 0.211–0.837) were associated with a reduced risk of developing IRP (Table S4).

### 3.3 Nomogram construction and validation

**FIGURE 3:**
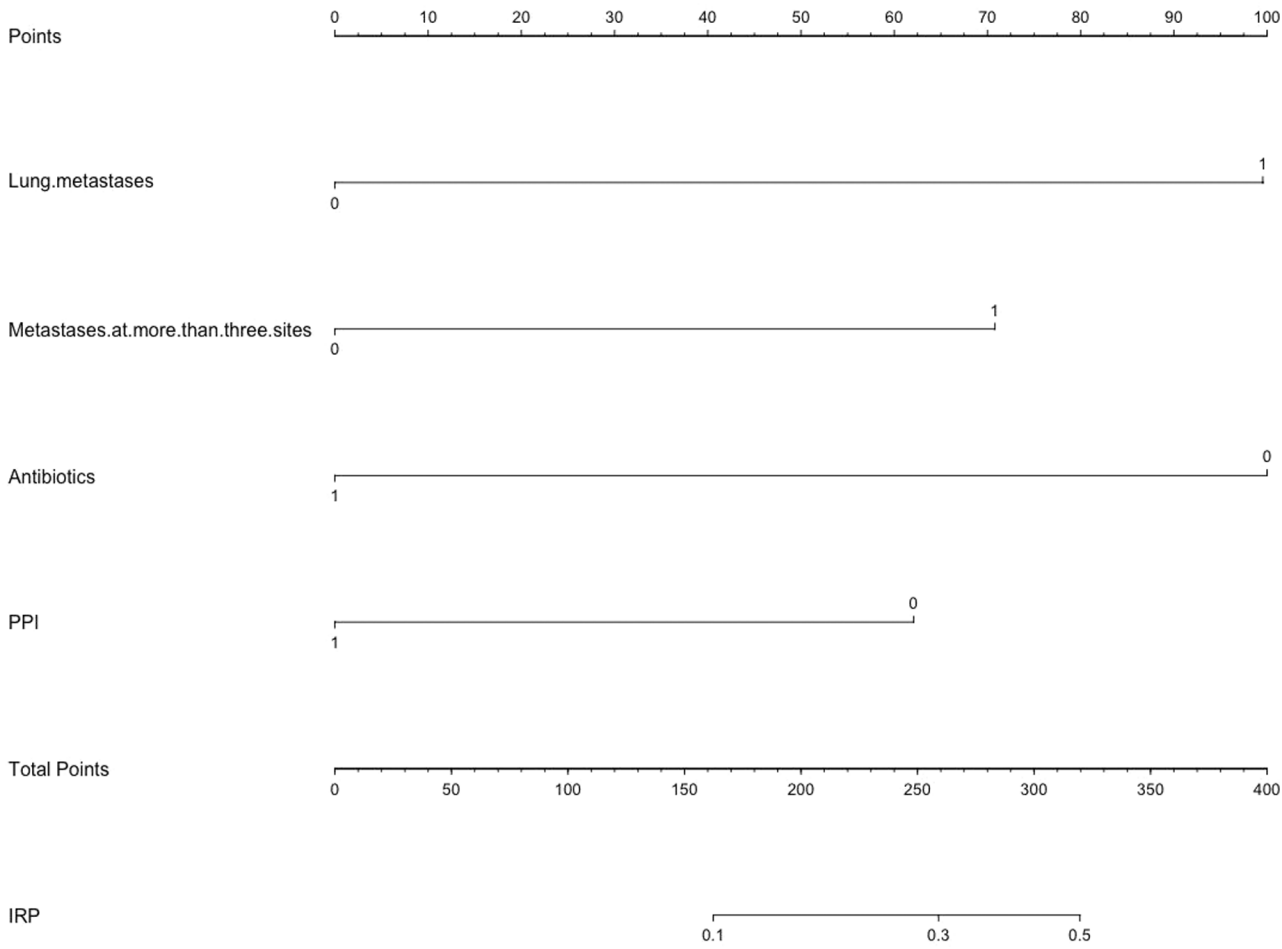
Developed nomogram. This nomogram was developed with metastases of the lung, metastases in at least three organ sites, use of antibiotics, and use of PPIs. IRP, immune‐related pneumonitis; PPI, proton pump inhibitor.

**FIGURE 4:**
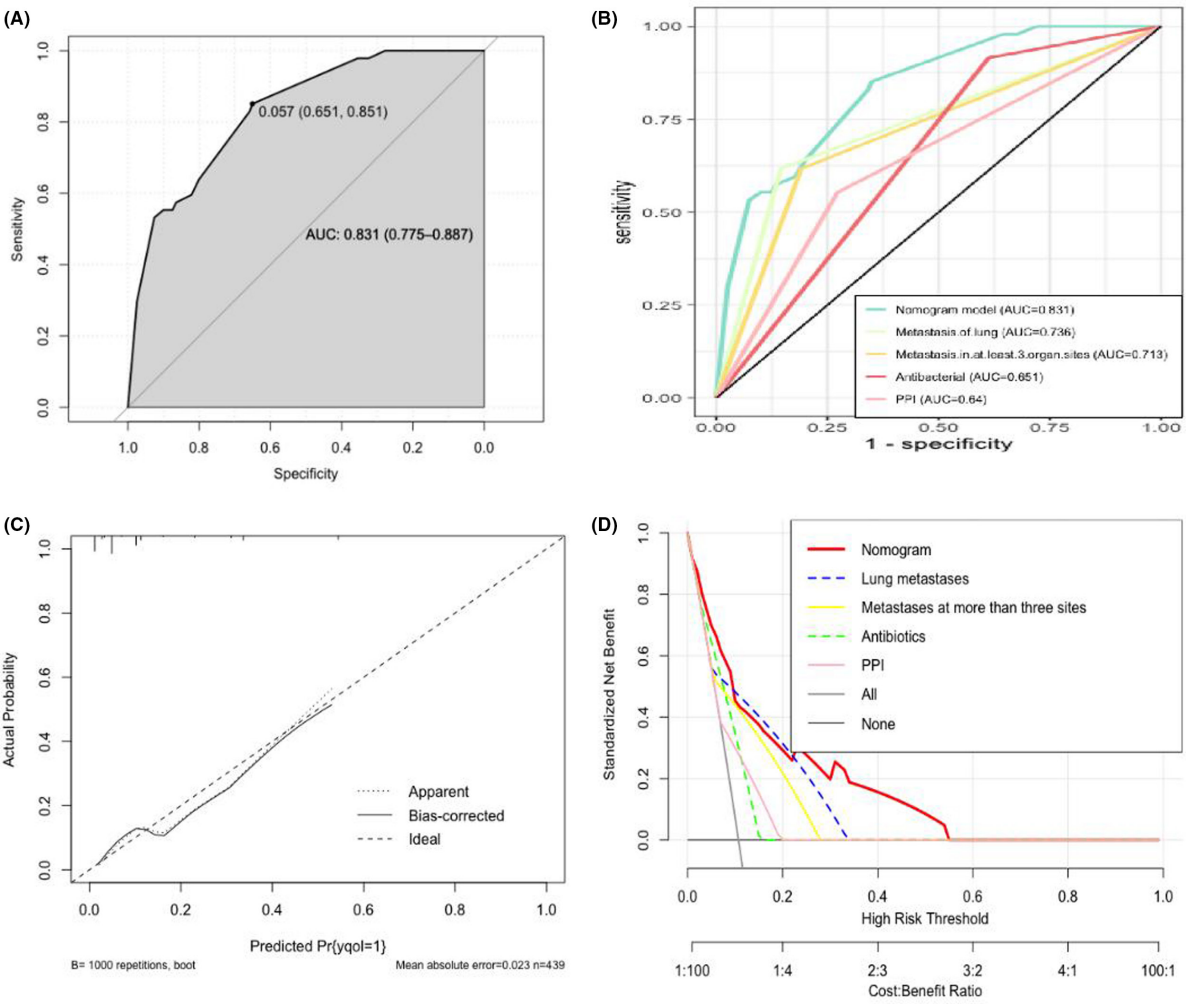
(A) Receiver operating characteristic (ROC) curve of the training set. The AUC was 0.831. (B) Comparison of the nomogram area under the curve (AUC) and single predictive factor AUC. (C) The calibration curve of the immune‐related pneumonitis (IRP) prediction model is represented by a solid black line. Dashed diagonal lines indicate an ideal nomogram model. (D) Analysis of the nomogram model (DCA). The y‐axis represents the net clinical benefit. The red line represents the line graph model that predicts IRP. The blue, yellow, pink, and green lines represent single‐factor predictors of IRP. AUC, area under the curve; DCA, decision curve analysis; IRP, immune‐related pneumonitis; PPI, proton pump inhibitor; ROC, receiver operating characteristic.

Logistic regression was used to generate a graph to predict the risk for developing IRP. Four clinical variables were included: lung metastases, metastases to at least three organ sites, combined antibiotic use, and combined PPI use (Figure 3). The nomogram had an AUC of 0.831 (95% CI: 0.775–0.887) for predicting the probability of IRP in suspected patients with IRP (Figure 4A,B). As shown in Figure 4C, the agreement between the predicted and actual probabilities was satisfactory. The *p*‐value of the Hosmer–Lemeshow goodness‐of‐fit test was 0.679, indicating that the model had good calibration (Table S5). The nomogram was validated against the validation set and complete datasets to evaluate the generalized performance of the final model. The predictive properties of the validation set were as follows: AUC = 0.876 (95% CI: 0.809–0.944), sensitivity = 100%, and specificity = 71.3%. Within the complete dataset, the final predictive properties of the model were as follows: AUC = 0.851 (95% CI: 0.806–0.895), sensitivity = 84.7%, and specificity = 72.8% (Figure S1). The probabilistic calibration curves for internal verification were satisfactory (Figure S2).

The DCA of the training set was compared with the curve based on the univariate model (Figure 4D). The nomogram had a large threshold probability range and greater net benefit than any single predictor. In the subgroup analysis, the nomogram achieved a very stable predictive performance in the following subgroups: age (<65 years and ≥65 years; AUC = 0.844 and 0.878, respectively), sex (AUC = 0.836 and 0.869 for females and males, respectively), cancer type (AUC of lung cancer, esophageal cancer, gastric cancer, lymphoma, intestinal tumor, liver cancer, and other tumors were 0.846, 0.827, 0.959, 0.943, 0.938, 0.748, and 0.816, respectively) (Table 2).

**TABLE 2:** ROC analysis in subgroups.

Subgroups	AUC
Age (years)	
≥65	0.878
<65	0.844
Sex	
Female	0.836
Male	0.869
Tumor type	
Lung cancer	0.846
Esophagus cancer	0.827
Gastric cancer	0.959
Lymphoma	0.943
Intestinal tumor	0.938
Liver cancer	0.748
Other tumors	0.816

Abbreviations: AUC, area under the curve; ROC, receiver operating characteristic.

## 4 DISCUSSION

This study presented an analysis of the incidence of IRP caused by sintilimab, identified independent risk factors for IRP in patients with tumors by analyzing patients undergoing sintilimab treatment, and developed and validated a nomogram to predict IRP. In addition, we stratified the nomogram according to sex, age, and cancer type to assess the predictive performance of the nomogram in different populations.

The incidence rate of IRP caused by sintilimab was 9.34%, which is greater than that reported in previous clinical trials.^5^, ^6^, ^7^ Due to the population in these clinical trials, study variables such as medication regimens and phases vary, which may limit the composition of accurate explanatory results. However, our data are consistent with other real‐world incidences.^8^, ^9^

Previous studies have shown that three or more tumor metastases are associated with a significantly increased risk of severe adverse events (AEs).^22^ This result may be due to interleukin 6, a core driver of tumor metastasis that endows the primary tumor cells (or the pre‐tumor cells that have not completely become malignant) with the ability to invade and metastasize, activates immune cells, and forms a positive feedback loop that causes a cytokine storm, resulting in the constant release of an array of inflammatory factors that leads to the appearance of related adverse symptoms.^23^, ^24^ Therefore, patients with three or more tumor metastases are at risk of developing IRP.

In addition, Uchida et al. found that lung metastasis is associated with an increased risk of developing IRP, which is consistent with our findings.^25^ The primary mechanism behind this association may be the recognition of exosome RNA secreted by tumors into the bloodstream by the natural immune receptor toll‐like receptor 3, expressed by type II lung epithelial cells. This recognition triggers the release of chemokines and other inflammatory responses, recruiting neutrophils to the lungs and establishing a localized inflammatory microenvironment within the lung tissue.^26^ Therefore, lung metastasis is closely associated with an increased risk of IRP.

Previous studies have shown that combining PPI and antibiotic treatment reduces the incidence of immune‐related AEs (irAEs).^27^ Our study yielded similar findings. However, the association between PPIs and antibiotics and the reduced risk of IRP is unclear. A possible explanation is that PPIs cause immunosuppression by reducing the expression of adhesion molecules in inflammatory cells or altering the secretion of pro‐inflammatory cytokines.^28^ PPIs also affect the gut microbiome by changing the pH of the stomach, delaying gastric emptying, and inducing the positive and negative selection of specific bacterial species in the gut.^29^, ^30^ However, the microbiome regulates the local immune response at the intestinal interface, and the bacterial peptide‐mediated distal T‐cell transport affects the toxicity patterns of ICIs.^31^ Antibiotics cause changes in the gut microbiome and have complex effects on the tumor‐host‐microbial interface by disrupting the gut ecosystem and causing downstream metabolic changes in the microenvironment. The catabolism of indigestible carbohydrates and the conversion of primary bile acids to *Clostridium* intermediate bile acids (including deoxycholic acid) result in changes in the availability of short‐chain fatty acids produced by bacteria such as *Faecalibacterium*.^32^ Emerging data suggest that certain strains, such as *Faecalibacterium*, are more likely to increase the risk of irAEs.^31^ Therefore, using PPIs and antibiotics reduces the risk of developing IRP. However, insufficient evidence shows that PPIs and antibiotics reduce IRP incidence and mortality. Although, given that chronic micro‐inhalation, including gastroesophageal reflux, may lead to lung injury, we recommend using PPIs for patients who have already been admitted. Guidelines for preventing stress ulcers should be followed for patients who do not use acid‐suppressive drugs at admission, and PPI prophylactic drugs should be preferred. Patients who are considered infected should be promptly treated with antibiotics.

Several predictive models have been developed for IRP. Although Jia et al. and Chao et al. created a nomogram of IRP based on patient history, clinicopathological features, and peripheral blood markers, a comprehensive analysis of related medication factors, such as past and combination medications, was lacking.^13^, ^14^ In addition, previous models have been based on small datasets. Therefore, the applicability of this model to other types of cancer is unclear. Therefore, we included a larger cohort, reassessed the risk factors, and created an IRP nomogram to incorporate them. Finally, four new variables were included. The AUC of the receiver operating characteristic (ROC) curve of the IRP nomogram was 0.831 (95% CI: 0.775–0.887) and was applied to different subgroups with good robustness. This nomogram model will help clinicians identify patients with a higher risk of developing IRP and carefully consider ICI treatment. Patients at a high risk of developing IRP and who must be treated with ICIs can be monitored more closely to prevent the progression of pulmonary toxicity.

This study had some limitations. First, different combinations of therapies (chemotherapy and targeted drugs) may lead to different susceptibilities to IRP owing to different pharmacological mechanisms. However, the sample size was insufficient to stratify the different combination treatments. Second, IRP is an exclusionary diagnosis, and competing diagnoses can be ignored. Third, this was a single‐center retrospective study, which may limit the applicability of our findings to cancer patient populations of other nationalities. Fourth, our research results only predicted their performance through internal verification, and we need to continue collecting external data for further verification. Finally, current studies have not fully clarified the pathogenesis of IRP, leading to a failure to fully consider the characteristics of the tumor and its microenvironment in the construction of the prediction model. With future improvements in understanding IRP pathogenesis, a more effective and comprehensive IRP prediction model should be established.

## 5 CONCLUSIONS

The incidence of IRP was higher in patients treated with sintilimab. The risk factors for IRP were lung metastasis and metastases at ≥3 sites, and the protective factor was treatment with a combination of PPIs and antibiotics. Our nomogram model may help clinicians assess the risk for development of IRP and develop optimal drug delivery strategies. By identifying patients at a high risk of developing IRP for clinical purposes, the morbidity and mortality of patients can be reduced. With further study on the pathogenesis of IRP, future genomic predictions of IRP occurrence may be possible.

## AUTHOR CONTRIBUTIONS

**BAOHUI HONG:** Data curation (equal); formal analysis (equal); methodology (equal); software (equal); supervision (equal); validation (equal); writing – original draft (equal); writing – review and editing (equal). **Rong Chen:** Data curation (equal); formal analysis (equal); methodology (equal); software (equal); supervision (equal); visualization (equal); writing – original draft (equal); writing – review and editing (equal). **Caiyun Zheng:** Formal analysis (equal); funding acquisition (equal); resources (equal). **Maobai Liu:** Data curation (equal); funding acquisition (equal); resources (equal). **Jing Yang:** Conceptualization (equal); data curation (equal); methodology (equal); writing – review and editing (equal).

## FUNDING INFORMATION

This study was supported by the Industry‐University Cooperation Fund of Fujian Province (Grant No. 2023Y41010075) and the Startup Fund for Scientific Research, Fujian Medical University (Grant number: 2020QH1346). However, the funder had no role in the design of the study; the collection, analysis, and interpretation of the data; or in writing the manuscript.

## CONFLICT OF INTEREST STATEMENT

The authors declare that they have no competing interests.

## ETHICS STATEMENT

Approval of the research protocol by an Institutional Reviewer Board. This study was approved by the Ethics Committee of Fujian Union Medical College Hospital (Approval number: 2021KY156).

## Supporting information


Data S1:

## Data Availability

All data generated or analyzed during this study are included in this published article and its supplementary information files. Further inquiries can be directed to the corresponding author.
